# Recanalization of complete biliary obstruction after liver transplantation with magnetosurgery

**DOI:** 10.1055/a-2849-9077

**Published:** 2026-04-27

**Authors:** Miaomiao Zhang, Yu Li, Ying Liu, Yanju Tong, Yi Lv, Xiaopeng Yan

**Affiliations:** 1 Department of Hepatobiliary Surgery 162798 The First Affiliated Hospital of Xiʼan Jiaotong UniversityXiʼanShaanxiChina; 2 Shaanxi Provincial Key Laboratory of Magnetic Medicine 162798 The First Affiliated Hospital of Xiʼan Jiaotong UniversityXiʼanShaanxiChina

**Fig. 1:**
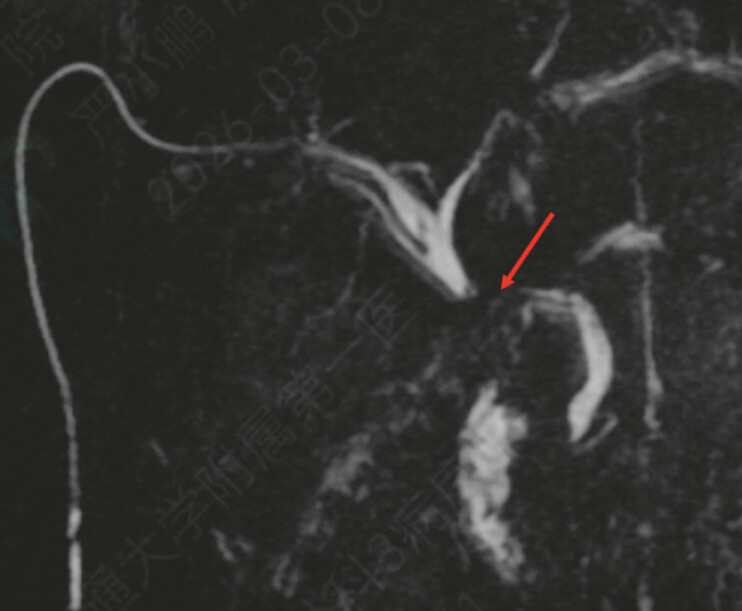
MRCP indicates obstruction of the upper segment of the common bile duct. MRCP, magnetic resonance cholangiopancreatography.

**Fig. 2:**
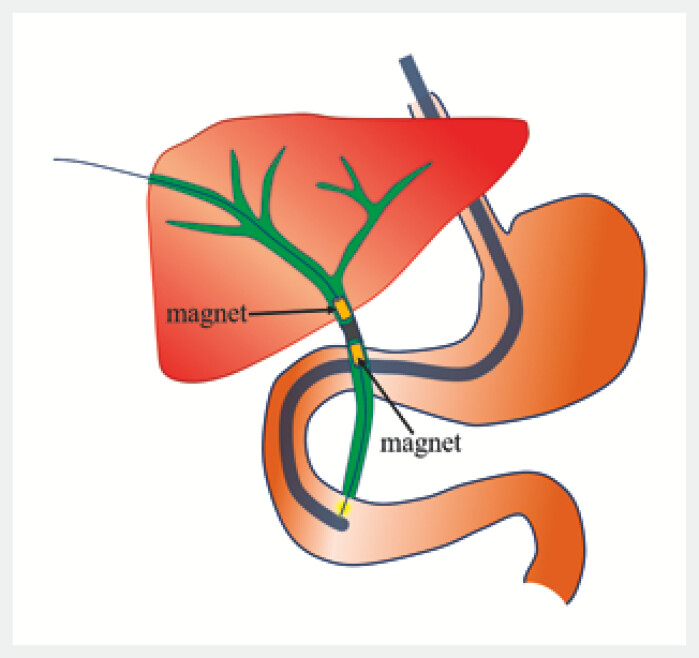
The surgical operation plan for recanalization of complete biliary obstruction using magnetosurgery.

A 59-year-old man, 6 months after liver transplantation for liver cancer, presented with a complete biliary occlusion at the anastomosis ( Fig. 1 ). Two weeks postoperatively, percutaneous transhepatic biliary drainage (PTBD) was performed due to bile leakage. However, endoscopic retrograde cholangiopancreatography (ERCP) confirmed that the occlusion was impassable via guidewire 3 months ago. The patient presented to our Magnetic Surgery Clinic for magnetosurgical intervention. After obtaining informed consent, a magnetosurgery procedure was planned ( Fig. 2 ).

**Fig. 3:**
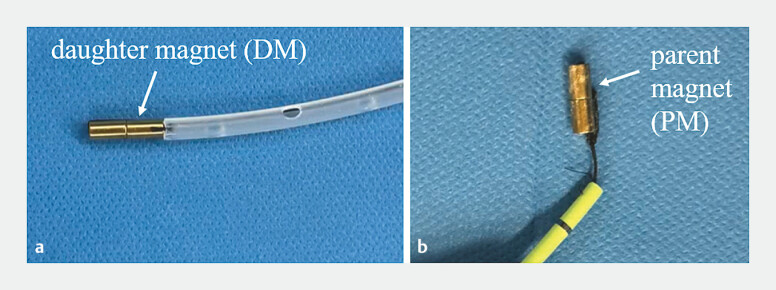
The magnets used during the surgery: **a** the daughter magnet (DM); **b** the parent magnet (PM).

**Fig. 4:**
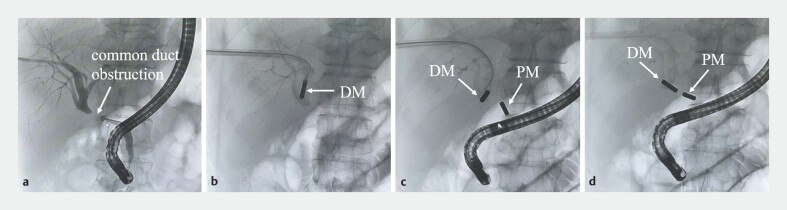
Placement of the magnets: **a** Cholangiography shows complete obstruction of the common bile duct; **b** the DM was inserted through the PTBD channel; **c** placing the parent magnets using ERCP; **d** the PM and DM attracted together. DM, daughter magnet; ERCP, endoscopic retrograde cholangiopancreatography; PM, parent magnet; PTBD, percutaneous transhepatic biliary drainage.

After anesthesia, combined cholangiography via PTBD and ERCP demonstrated complete discontinuity between the intrahepatic and extrahepatic bile ducts. A guidewire was advanced through the PTBD tract, followed by sinus tract dilation using a 16 F sheath. A 5-mm daughter magnet (DM, Fig. 3 **a** ) was inserted via the PTBD tract to the proximal end of the biliary obstruction, while a 5-mm parent magnet (PM, Fig. 3 **b** ) was delivered distally via the duodenal papilla using ERCP. The magnets were adjusted until they automatically attracted ( Fig. 4 ). External biliary drainage was maintained postoperatively.

**Fig. 5:**
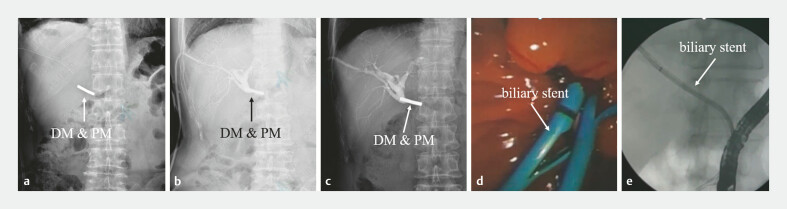
Postoperative magnet status and the placement of the biliary stent: **a** POD 1, **b** POD 13, and **c** POD 29; **d** after removing the magnets, a biliary stent was placed under ERCP; **e** the biliary stent as seen on X-ray. ERCP, endoscopic retrograde cholangiopancreatography; POD, postoperative day.

On postoperative day (POD) 1, X-ray confirmed the stable position of the magnets. Follow-up cholangiography via the PTBD tube on POD 13 and POD 29 revealed no visualization of the distal bile duct. On POD 29, a guidewire was inserted through the PTBD tract while the DM was being withdrawn by pulling the attached line. Further advancement of the guidewire into the distal bile duct facilitated the displacement of the PM, which was then successfully retrieved via a basket under ERCP. Following balloon dilation, a biliary stent was successfully deployed ( Fig. 5 and Video 1 ), and the external drainage tube was completely withdrawn. No adverse events occurred during or after the procedure. Over a year of follow‑up, the biliary stent has been removed and the patient is currently in good condition.

The surgical procedure of magnetosurgery.Video 1

The management of complete biliary obstruction after liver transplantation remains clinically challenging. Magnetosurgery has demonstrated favorable outcomes in treating esophageal stenosis 1 2 as well as colorectal stenosis and obstruction 3 4 . Additionally, a recent study indicated that magnetosurgery was an effective non-surgical treatment option for patients with completely occluded refractory benign biliary strictures 5 . Consequently, magnetosurgery holds significant clinical potential for addressing benign biliary obstructions and warrants further attention.

Endoscopy_UCTN_Code_TTT_1AR_2A
